# Comparison of modified wet suction technique and dry suction technique in endoscopic ultrasound-guided fine-needle aspiration (EUS-FNA) for solid lesions: study protocol for a randomized controlled trial

**DOI:** 10.1186/s13063-017-2380-y

**Published:** 2018-01-17

**Authors:** Yun Wang, Qian Chen, Jinlin Wang, Xiaoli Wu, Yaqi Duan, Ping Yin, Qiaozhen Guo, Wei Hou, Bin Cheng

**Affiliations:** 1 Department of Gastroenterology and Hepatology, Tongji Hospital, Tongji Medical College, Huazhong University of Science and Technology, Wuhan, 430030 China; 2 Department of Pathology, Tongji Hospital, Tongji Medical College, Huazhong University of Science and Technology, Wuhan, 430030 China; 3 Department of Epidemiology and Biostatistics, School of Public Health, Tongji Medical College, Huazhong University of Science and Technology, Wuhan, 430030 China

**Keywords:** EUS-FNA, Modified wet suction technique, Dry suction technique, Solid lesions, Diagnostic yield

## Abstract

**Background:**

Several suction techniques have been developed recently to enhance tissue acquisition when sampling solid lesions using endoscopic ultrasound-guided fine-needle aspiration (EUS-FNA). The aim of this study is to determine whether a new modified wet suction technique (MWST) compared with the conventional dry suction technique (DRST) shall present better outcomes with respect to diagnostic yield and specimen quality of solid lesions in the intra-abdomen and mediastinum.

**Methods/design:**

This is a single-blind, randomized, controlled, superiority trial conducted at four large tertiary care centers in China. Two hundred and ninety-six patients with solid lesions referred for EUS-FNA will be randomly assigned to group A, using DRST for the first pass, or group B, using MWST for the first pass in a ratio of 1:1. Following a 2 × 2 cross-over design, the pass sequence for group A is DRST, MWST, DRST, MWST. For group B, the pass sequence is MWST, DRST, MWST, DRST. All procedures will be performed by experienced echoendoscopists, and the patients and assessors (cytologists and pathologists) will be blinded during the entire study. The primary outcome measure is the diagnosis yield. Secondary outcome measures are specimen quality, including assessment of quantity of cell, tissue integrity, and blood contamination.

**Discussion:**

To our knowledge, this is the first large-scale randomized controlled trial to compare MWST with DRST when sampling solid lesions in the intra-abdomen and mediastinum. The results may contribute to future multicenter clinical trials in standardizing suction techniques during EUS-FNA.

**Trial registration:**

Clinical Trials.gov, NCT02789371. Retrospectively registered on 6 June 2016.

**Electronic supplementary material:**

The online version of this article (doi:10.1186/s13063-017-2380-y) contains supplementary material, which is available to authorized users.

## Background

Endoscopic ultrasound-guided fine-needle aspiration (EUS-FNA) is now a fundamental tool in cytopathological and histopathological diagnosis, and a variety of clinical trials have been conducted to further optimize the diagnostic yield of FNA [1–8]. Among those, the role of suction during EUS-FNA remains unclear and has not been standardized [9, 10].

The standard suction technique utilizes a needle controlled under negative pressure, usually applied with a 10 ml syringe; the advantage of negative pressure is an increase in cellularity. As the conventional dry suction technique (DRST) is conducted by applying negative pressure suction at the proximal end of the needle after the stylet is removed with an air-filled pre-vacuum syringe, this maneuver may also cause blood contamination and thus hinder accurate cytological interpretation [11]. To overcome this issue, the wet suction technique was developed, relying on pre-flushing the needle with saline to replace the column of air with fluid followed by aspiration at the proximal end, using a prefilled suction syringe with saline. It has been suggested that the presence of a saline-solution column might keep the needle from getting clogged while avoiding the inherent inconvenience of a metal stylet [12]. There was a single-blind and randomized trial on 117 patients showing that wet suction technique in comparison with DRST indeed yielded significantly higher cellularity (1.82 ± 0.76 vs. 1.45 ± 0.768, *P* < 0.0003) and better specimen adequacy for obtained cell-block (85.5% vs. 75.2%, *P* < 0.035) [12]. No difference in the amount of blood contamination between the two techniques was observed in that study.

Following the same principle that preloading a needle with water, which is a less compressible fluid than air, may enhance tissue aspiration through increasing the volume of vacuum forced to the distal tip of the needle, a new modified wet suction technique (MWST) was recently developed [13, 14]. The MWST consists of preparing the needle as with the wet technique but applying suction as with the dry technique by having continuous negative pressure through a pre-vacuum syringe to avoid manual intermittent suction [13]. A preliminary result from a single-center study of 15 patients highlighted that MWST achieved a larger amount of volume aspirate than DRST when sampling solid lesions in the intra-abdomen and mediastinum. However, for both sample adequacy and final diagnosis, there was no significant tendency in favor of pre-filling the needle with normal saline (both MWST and the wet suction technique) compared with a standard (DRST) technique; perhaps the effect was not detected in this underpowered pilot study [13].

The objective of this randomized controlled trial is to evaluate whether MWST, compared with DRST, shall present a better outcome with regard to the diagnostic yield and specimen quality of patients with solid lesions in the intra-abdomen and mediastinum. All patients will undergo MWST and DRST in alternating fashion following a cross-over design.

## Methods/design

This is a multicenter, single-blind, randomized, controlled, cross-over, superiority trial. The SPIRIT Checklist is provided as Additional file 1. A total of 296 patients will be enrolled from endoscopic centers at four large tertiary hospitals, including Tongji Hospital, Union Hospital, Renmin Hospital of Wuhan University, and the Central Hospital of Wuhan.

**Fig. 1:**
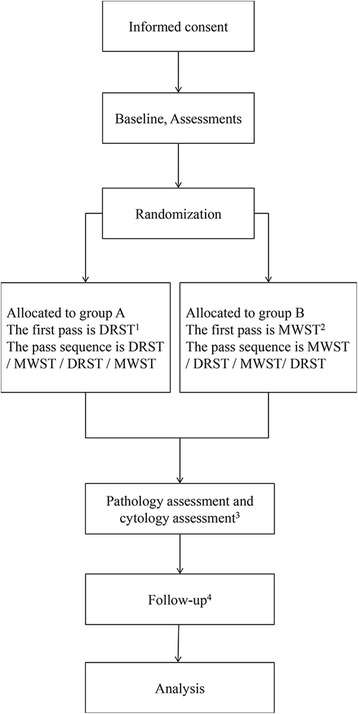
CONSORT flowchart illustrating the randomization and recruitment process in the study. ^1^ DRST, dry suction technique. ^2^MWST, modified wet suction technique. ^3^ Each slide will be assessed by two independent experts. The cytologists and pathologists will follow the protocol to assess the samples and will be blinded as to which technique was used for which specimen. ^4^ Four follow-up points are scheduled after the biopsy (1, 12, 24, and 48 weeks after the operation); thereafter, follow-ups will be conducted via telephone interviews or outpatient interviews. Once the surgical pathologic results are obtained, we will stop the follow-up sessions.

All patients will be examined with a 22G echo tip ultra needle for four passes. Patients will be divided into group A and group B in a 1:1 ratio. For group A, the pass sequence is DRST, MWST, DRST, MWST . For group B, the pass sequence is MWST, DRST, MWST, DRST. The flowchart in Fig. 1 illustrates the recruitment process.

### Ethics

The study was approved by the ethics committee of Tongji Medical College, Huazhong University of Science and Technology (IORG no., IORG0003571) on 4 May 2016. Subsequently, the boards of the three participating hospitals gave permission to conduct the trial. Informed consent will be obtained from each participant or from each participant’s legally responsible relative.

The trial was registered at Clinical Trials.gov, NCT02789371.

### Primary endpoint

The primary endpoint is to compare the use of MWST with that of DRST on the overall diagnosis yield of solid lesions in the intra-abdomen and mediastinum.

### Secondary endpoints

The secondary endpoints are as follows:

1. The diagnostic yield, including cytological and histological assessment for benign solid lesions
2. The diagnostic yield, including cytological and histological assessment for malignant solid lesions
3. The diagnostic yield, including cytological and histological assessment for pancreatic solid lesions
4. The diagnostic yield, including cytological and histological assessment for non-pancreatic solid lesions
5. The degree of cellularity for specimens obtained using MWST and DRST
6. Blood contamination of specimens obtained using MWST and DRST

### Inclusion criteria

The inclusion criteria are as follows:

1. Age > 18 years, < 85 years
2. Male or female
3. Patients referred for EUS-FNA after imaging examination (MRI, CT, or ultrasonography) that shows either mediastinal, pancreatic, or non-pancreatic intra-abdominal, or pelvic cavity solid lesions (size > 1 cm)
4. Signed informed consent letter

### Exclusion criteria

The exclusion criteria include the following:

1. Hemoglobin ≤ 8.0 g/dl
2. Pregnant
3. Coagulopathy (platelet count < 50,000/mm^3^, international normalized ratio > 1.5) or having taken oral anticoagulation agents such as aspirin or warfarin in the previous week
4. Acute pancreatitis in the previous 2 weeks
5. Severe cardiorespiratory dysfunction
6. Psychiatric disease, drug addiction, or other reason for unreliable follow-up or questionnaires
7. Absence of informed consent

### Randomization and blinding

The randomization list (nos. 001 to 296) was generated by an independent statistician who was blinded to this study using SAS 9.2 to generate randomized block (block size = 8). Scratch cards are used to ensure that the patients will be divided into group A (*n* = 148) and group B (*n* = 148) in a ratio of 1:1. After baseline assessment and the provision of informed consent by patients, only the echoendoscopists will know which suction technique to use by scratching off the card during the procedure, whereas the patients and assessors (cytologists and pathologists) will be blinded during the entire study.

### EUS-FNA procedure

The procedure will be performed under deep sedation according to the principles of “monitored anesthesia care”. The patients will be anesthetized with intravenous administration of propofol by anesthetists. All patients will receive oxygen during the procedures; blood pressure and heart rate will be monitored. Procedures will be performed by one of eight experienced echoendoscopists (Bin Cheng, Xiaoli Wu, Ding Zhen, Liangru Zhu, Shiyun Tan, Jiwang Cao, Gan Shi, Jian Wang) from the four centers (each with experience performing > 1000 EUS procedures). A linear Olympus echoendoscope (GF-UCT 260, GF-UCT 240; Olympus, Tokyo, Japan), a Fujifilm linear echoendoscope (EG-530UT2; Fujifilm, Tokyo, Japan) and a Pentax linear echoendoscope (EG 3870UK, EG 3270UK; Pentax, Tokyo, Japan) are used. After the solid lesion in the mediastinum or intra-abdomen is identified by the echoendoscopist under real-time EUS, the FNA needle (22G Echo Tip Ultra needle, Cook Medical) will be advanced within the lesion and a total of four needle passes will be performed. The pass sequence for group A is DRST, MWST, DRST, MWST, and the pass sequence for group B is MWST, DRST, MWST, DRST.

### Dry suction technique

In the DRST, prior to puncturing the lesion, the stylet is removed and a 5 ml air-filled pre-vacuum syringe is attached to the needle in a “locked” position [15]. The needle is moved back and forth 20 times by applying negative pressure suction within the lesion. Afterwards, the needle is withdrawn from the lesion.

### Modified wet suction technique

In the MWST, prior to puncturing the lesion, the stylet is removed and needle is pre-flushed with 1–2 ml of saline using a 10 ml syringe in order to replacing the column of air with fluid. After the needle punctures the lesion, the suction syringe will be replaced with a 5 ml air-filled pre-vacuum syringe, which applies continuous negative pressure suction while the needle moves back and forth 20 times within the lesion [13].

### Specimen assessment

After FNA tissue acquisition, each specimen will be mounted onto a slide by inserting the stylet, flushing the needle with 0.1 ml sterile saline, and further applying air flush [16]. The specimen will be carefully examined for the presence of a macroscopic visible core, which is defined as whitish or yellowish pieces of tissue with an apparent bulk, not including paste-like or liquid-like specimens [17]. For histological analysis, the macroscopic visible core and blood clots, if present, will be picked up from the slide using surgical tweezers and then transferred into Eppendorf tubes containing formalin. The remaining specimen on the slide will be used in smear for cytologic evaluation.

All the samples will be assessed for adequacy on site by the endosonographer and the endosonographer’s assistant, who have been trained for macroscopic onsite evaluation previously [17], and subsequently confirmed by an experienced cytopathologist at each center. Each pass will be assessed immediately for cellular adequacy. If no macroscopic visible core is obtained, the echoendoscopist will conduct a backup procedure through switching to either MWST or DRST for an additional number of passes based, using his or her own judgment, to obtain adequate specimen.

Cytologists and pathologists from Tongji Hospital, Tongji Medical College, Huazhong University of Science and Technology, will reassess all samples every 3 months following the protocol and remain blinded regarding which technique was used. Each sample will be assessed by two independent experts. If the two experts make different judgments, they should review all the clinical materials together to make a final decision [4].

Criteria for cytological assessment include quantity of cells [18] and blood cell contamination [15], as follows:

1. Quantity of cells:
  Grade A. Satisfactory, have > 4 clusters for adequate cytological interpretation with a minimum of 6–8 cells per cluster; or have structure-clear nucleated cell counts > 200
  Grade B. Adequate, have 2–4 clusters for adequate cytological interpretation with a minimum of 6–8 cells per cluster; or have structure-clear nucleated cell counts of 50–200
  Grade C. Unsatisfactory, have < 2 clusters for adequate cytological interpretation or probably not representative; or have structure-clear nucleated cell counts < 50
2. Blood cell contamination:
  Grade A. Minimal contamination, blood cells present in < 1/4 of the slide
  Grade B. Moderate contamination, blood cells present in 1/4–1/2 of the slide
  Grade C. Significant contamination, blood cells present in > 1/2 of the slide

Criteria for histologic assessment include tissue integrity and blood contamination [19, 20], as follows:

1. Tissue integrity assessment:
  Grade A. The presence of a tissue core (defined as an architecturally intact piece of tissue measuring at least 550 μm in greatest axis), is sufficient for making a diagnosis
  Grade B. Core fragments are present: tissue does not meet the criteria for architecturally intact histology but can still yield a diagnosis based on cell morphology
  Grade C. No architecturally intact tissue is present, and cannot yield diagnosis
2. Blood contamination assessment:
  Grade A. Minimal contamination, blood cells present in < 1/4 of the slide
  Grade B. Moderate contamination, blood cells present in 1/4–1/2 of the slide
  Grade C. Significant contamination, blood cells present in > 1/2 of the slide

### Data collection

**Fig. 2:**
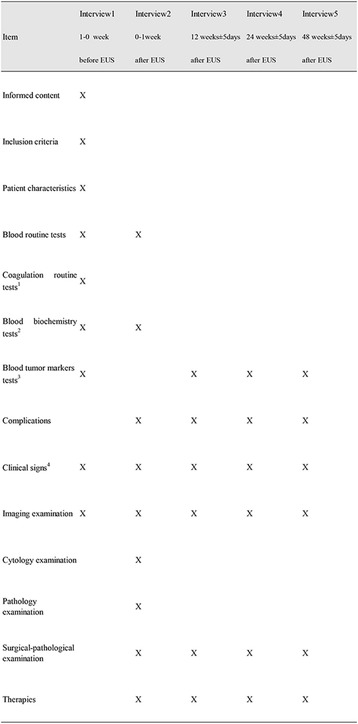
Standard protocol items (SPIRIT): schedule for data collection. ^1^Coagulation routine tests: prothrombin time, activated partial thromboplastin time, thrombin time, fibrinogen, international normalized ratio. ^2^Blood biochemistry tests: aspartate aminotransferase, alanine aminotransferase, blood urea nitrogen, creatinine, alkaline phosphatase, lipase, amylase. ^3^Blood tumor markers tests: carcinoembryonic antigen, carbohydrate antigen 19-9, carbohydrate antigen 72-4, alpha fetal protein, squamous cell carcinoma antigen, neuron-specific enolase. ^4^Clinical signs include pain, weight loss, cachexia, etc. EUS, endoscopic ultrasound

Paper case report forms will be used and a standard operating procedure for case report form entry will be prepared (Fig. 2) [16].

### Final diagnosis and diagnostic criteria

For histological and cytological evaluation, diagnosis will be classified into four categories: “positive for malignancy”, “suspicious for malignancy”, “atypia”, and “negative for malignancy” [21].

The diagnostic criteria are based on the following [19]:

1. For patients who undergo surgery, the final diagnoses will be based on the definite histological diagnoses from surgical resection specimens.
2. In the absence of surgical pathology, a minimum 48 weeks of clinical follow-up time will be conducted. If the lesion spontaneously resolves or has no sign of deterioration in follow-up imaging studies, the lesion will be considered a benign disease. If the lesion shows enlargement or metastasis, and the patient presents malignant symptoms such as weight loss, anemia, or dies of tumor complications during the follow-up, the lesion will be considered a malignant disease.

### Sample size and statistical analysis

Based on a literature review of suction techniques for heterogeneous indications [12, 14], we expected a difference between DRST and wet suction technique on diagnostic yields of 75% vs. 85% after four needle passes. The calculation yielded target sample sizes of 248 for 5 ml DRST and 248 for MWST, with a power of 80% and a two-sided significance level of 5%. Each group should have 124 patients following the 2 × 2 cross-over design. Assuming a 20% dropout or withdrawal rate, we calculated a final sample size of 296 patients (148 per group).

The safety set comprises all the randomized subjects who will receive at least one trial treatment. The full analysis set should be as close as possible to the intention-to-treat set. The standards and population of the per-protocol set will be finalized after data blinding verification. The direct deletion method will be used to treat missing data.

A two-tailed distribution will be used and statistically significance will be considered for *P* < 0.05. All category variables will be described in terms of the count and percentage using the *χ*^2^ test, whereas continuous variables will be described as mean ± standard deviation using *t* tests or Wilcoxon rank-sum tests. Sensitivity, specificity, positive predictive value, negative predictive value, receiver operating characteristic curve, and diagnostic accuracy will be computed using a 2 × 2 table and *χ*^2^ test for DRST and MWST. The blood contamination and cellularity in specimens will be divided into three levels (Grade A, Grade B, and Grade C), and McNemar’s test for correlated proportions will be used. All analyses will be performed using SAS version 9.2.

## Discussion

Currently, poor cellularity of the aspirates obtained through EUS-FNA is a common cause of the lack of a diagnosis, which may result in repeated procedures and a delay in reaching a diagnosis [22, 23]. Dry and wet suction techniques were developed to improve the performance of aspirating solid lesions in the intra-abdomen and mediastinum, but neither has yet been recommended as a standardized EUS-FNA suction technique. Applying the DRST is related to more cellularity during tissue acquisition but more blood contamination may occur and thus affect the overall quality of the specimen [18]. By studying a three-dimensional computational fluid dynamic model, it has been suggested that because water is a less compressible fluid than air a water-filled needle (wet technique) is superior to an air-filled needle, as it allows faster aspiration of material into the distal end of the needle [13]. A recent randomized controlled trial by Attam *et al.* [12] compared the wet suction technique with the DRST among 117 patients and favored the wet suction technique for tissue acquisition to obtain a better diagnostic yield; however, it was not able to conclude on the difference in blood contamination between the two techniques [12, 13].

A hybrid suction technique, MWST, is a new way of applying negative suction pressure during EUS-FNA in a manner that combines the wet technique for needle preparation and the dry technique for syringe preparation. Recent reports by Berzosa *et al.* [14] compared all three suction methods among 15 patients and failed to show favorable results for MWST. Nevertheless, from the clinical practice aspect, Villa *et al.* [13] indeed recommends MWST over the wet suction technique and DRST, given its simplicity and efficiency.

Here, we carry out a randomized controlled trial to compare the diagnostic yield and specimen quality between MWST and DRST. To our knowledge, this is the largest multicenter randomized controlled trial designed to apply MWST for diagnosing solid lesions in the intra-abdomen and mediastinum. The findings will help us optimize the sampling techniques and furthermore determine the most suitable technique for various solid lesions. Additionally, in our design, each participant will undergo both sampling techniques, thereby eliminating bias.

Some limitations exist in the present study design. First, rapid onsite evaluation is still not feasible among the majority of the endoscopy centers in China. The absence of rapid onsite evaluation may lead to an increase in the number of inadequate samples and thus may affect the diagnostic yield. However, all the samples will be assessed for adequacy on site by the endosonographers and their assistants, who have been trained for macroscopic onsite evaluation previously [17]. Secondly, not all patients would have a final pathology report through surgery, including patients with benign diseases, patients who have malignant lesions but lost the opportunity of undergoing operation, as well as patients who refuse surgical treatment. Thus, a minimum of 48 weeks of clinical follow-up will be conducted to help make the final diagnosis. To prevent dropout during follow-up, we will carefully monitor the recording process and ensure that fully informed consent is obtained and complete registration information recorded, and furthermore to record at least two contact phone numbers for each patient.

## Trial status

Patient enrollment began on 18 May 2016 and completion is expected by 31 September 2018. At present (22 April 2017), 152 patients have been enrolled in the study.

## Additional file

Additional file 1: SPIRIT 2013 checklist. (DOC 124 kb)

## Abbreviations

CT: Computed tomography
DRST: Dry suction technique
EUS: Endoscopic ultrasonography
EUS-FNA: Endoscopic ultrasound-guided fine-needle aspiration
FNA: Fine-needle aspiration
MRI: Magnetic resonance imaging
MWST: Modified wet suction technique
SAS: Statistical Analysis System
